# Physical (in)activity, and its predictors, among Brazilian adolescents: a multilevel analysis

**DOI:** 10.1186/s12889-021-12336-w

**Published:** 2022-02-03

**Authors:** Thayse Natacha Gomes, Mabliny Thuany, Fernanda Karina dos Santos, Thomas Rosemann, Beat Knechtle

**Affiliations:** 1 Department of Physical Education, Federal University of Sergipe, São Cristóvão, SE Brazil; 2 CIFI2D, Faculty of Sports, University of Porto, 4200-450 Porto, Portugal; 3 Department of Physical Education, Federal University of Viçosa, Viçosa, MG Brazil; 4 Institute of Primary Care, University of Zurich, 8091 Zurich, Switzerland; 5 Medbase St. Gallen Am Vadianplatz, Vadianstrasse 26, 9001 St. Gallen, Switzerland

**Keywords:** Adolescents, Guidelines compliance, Physical activity, School

## Abstract

**Background:**

Physical activity is a multifactorial trait, determined by both individual and environmental characteristics, it seems relevant to understand the determinants related to youth guidelines accomplishment. The present study aimed to verify the differences between the Brazilian federative units regarding to the prevalence of youth physical activity guidelines accomplishment and to investigate the determinants related to the inter-individual differences in this accomplishment.

**Methods:**

Sample comes from the 2015 Brazilian National School Health Survey (PeNSE), comprising 99,570 adolescents (51,527 girls, 13-17y), enrolled in 3039 schools. Adolescents reported the time they spend in moderate-to-vigorous physical activity daily, and they were categorized as active or inactive, if the guidelines were achieved, or not, respectively, and multilevel statistical analyses were used, including both child and school-level variables. Multilevel Binomial model was computed in the SuperMix software.

**Results:**

The majority of the adolescents did not comply with the physical activity guidelines daily, where Bahian children complied the least, while those from Amazonas, Tocantins, and Mato Grosso do Sul complied the most. Boys (OR: 2.305; 95%CI: 2.277-2.334), older adolescents (OR: 1.044; 95%CI: 1.036-1.051), and those who spent more time in active travelling to/from school (OR: 1.001; 95%CI: 1.001-1.001) complied more the physical activity guidelines. At the school level, adolescents from larger schools (OR: 0.957; 95%CI: 0.928-0.986) tended to comply less with the guidelines.

**Conclusion:**

Significant differences between Brazilian federative units in youth daily physical activity guidelines compliance were observed, highlighting the role of individual but also environmental constraints in the Brazilian adolescents’ engagement in physical activity.

## Background

In the last years, increases in physical inactivity have been pointed as one of the most relevant public health problems, especially among youth, given the health problems related to it such as non-communicable diseases [1]. Taking this into account, the World Health Organization (WHO) suggests that, for health benefits, children and adolescents should engage in, at least, 60 min of moderate-to-vigorous physical activity (MVPA) daily [2]. However, international data has pointed that 81.0% of adolescents are insufficiently active [3], and in the Brazilian context, a survey performed by the Brazilian Institute of Geography and Statistics (IBGE), reported that 46.4% of adolescents aged 15-17 years do not engage regularly in physical activity practices [4].

Given that Brazilian states show a great diversity between them, regarding not only to cultural aspects but also regarding economic and social factors [5], these differences may reflect in the youth physical activity guidelines accomplishment. For example, as reported by Cureau et al. [6] there is a diversity in the proportion of inactive adolescents across Brazilian regions, and these prevalences differ between sex, age groups, ethnicity, and socioeconomic status. In addition, studies focusing in the identification of determinants of youth physical activity, have pointed that a large number of predictors can act in the explanation of the inter-individual variability in physical activity levels, which are related to individual and also to environmental characteristics, such as age, sex, sociodemographic, socioeconomic status, parental education, school infrastructure, and physical education classes [7–11].

Given that, since physical activity is a multifactorial trait, determined by both individual and environmental characteristics, it seems relevant to understand the determinants related to youth guidelines accomplishment. Thus, the present study aims (1) to verify the differences between the Brazilian federative units regarding to the prevalence of youth physical activity guidelines accomplishment and (2) to investigate the determinants related to the inter-individual differences in this accomplishment.

## Methods

### Sample

The sample of the present study comes from a cross-sectional study conducted by the Brazilian Institute of Geography and Statistics (IBGE) in association with the Ministry of Health. Data used is from the 2015 Brazilian National School Healthy Survey (PeNSE), an epidemiological study carried out with Brazilian school-children enrolled in both public and private schools, from the 26 states capitals and the Federal District, as well as from other municipalities which were grouped into 26 geographical strata (representing each one of the Brazilian states, excluding their capitals) (more details about PeNSE design, see Oliveria et al. [12]). Given that, in this study, it was used information from 9th grade adolescents, aged 13-17 years, in a total of 99,570 subjects (51,527 girls, 48,043 boys), enrolled in 3039 schools. The PeNSE was approved by the National Committee of Ethics in Research (CONEP) (reports N° 11.537/2009, N° 16.805/2012, and N° 1.006.467/2015), meeting the Resolution of the National Health Council (N° 196, October/1996), following the Helsinki Declaration on human subjects testing.

### Physical activity

Information regarding daily time spent in physical activity, as well as time spent in active commuting to/from school were self-reported via questionnaire [13], which was developed to achieve PeNSE’s purpose. Students were asked about the time they spend in physical activity/day, and were categorized as active or inactive, if they reported to get engaged, or not, respectively, in at least 60 min/day in physical activities. Then, the number of days youth comprised the daily 60 min of physical activity were summed, and this variable was the one used in the analysis (which ranged from 0 to 7 days).

Furthermore, they were also asked to inform how many minutes per week they spent in active commuting to/from school, and the total weekly amount was used in the present study.

### Maternal education level

Adolescents answered about the educational level of their mothers, ranging from “did not study” until “undergraduate or higher level”.

### School context

Information about the school environment was obtained via questionnaire, answered by the school’s principals or his/her designee. From the set of available variables, those chosen from the study were “school size”, “the existence of gymnasium”, “the existence of playground area”, and “the existence of sports equipment”, based on previous studies where these variables were used as possible predictors of physical activity in school-children [14, 15]. All these variables were dichotomized.

### Statistical analysis

Descriptive statistics are presented as mean and standard deviation, or frequencies, which were computed in the SPSS 24. Since the dependent variable (number of days adolescents comply with at least 60 min/day in physical activities) is a count ranged from 0 to 7, and the hierarchical structure of the data, a multilevel Binomial model was used, computed in the SuperMix v.1 software, and using a full maximum likelihood approach. The modelling was performed in three steps: Model 1, including only the federative units. To observe federative units differences in physical activity compliance days, federative units were dummy coded, where the Bahia state was the site reference (since it showed the lowest percentage of children classified as active); Model 2, adding the child-level predictors - sex (girls were the reference), age (mean centred), time spent in commuting transport to/from school (mean centred), and maternal education level (which was dummy coded, and “did not study” was the reference category); and Model 3, including the school-level predictors - “school size”, “the existence of gymnasium”, “the existence of playground area”, and “the existence of sports equipment”, where all the school-level predictors were dichotomized (except for the variable “school size”, where the reference category was < 500 students, for all the other variables the reference category was “does not exist/unusable” gymnasium, playground area, and sports equipment). Significance level was set at 5%.

## Results

Descriptive statistics for child and school-level variables are provided in Tables 1 and 2, respectively, per federative units. Subjects mean age was about 14.3 ± 0.9 years and adolescents reported to spend, in mean, between 74 min and 100 min/week in active travel to/from school, highlighting that federative units from North and Northeast regions were those that showed the smallest and the highest values, respectively. Regarding the mother instruction, in all states the highest percentage was observed for the option “high school or incomplete undergraduate level”.

**Table 1:** Descriptive information [mean(standard deviation) or percentage] of child-level variables, per federative units

Region	Federative units	Age (years)	Travel time (min)	Mother instruction (%)			
				Did not study	Elementary school or incomplete high school	High school or incomplete undergraduate	Undergraduate or higher level
North	Rondônia	14.47(0.95)	84.66(115.81)	23.5%	10.2%	24.9%	18.2%
	Acre	14.16(0.93)	104.16(136.40)	27.3%	11.4%	18.8%	15.4%
	Amazonas	14.38(0.94)	88.41(125.81)	21.7%	12.6%	24.4%	16.1%
	Roraima	14.39(0.97)	86.36(126.14)	22.0%	12.1%	22.1%	15.8%
	Pará	14.36(1.03)	91.93(117.05)	19.9%	14.5%	27.7%	14.2%
	Amapá	14.24(1.01)	90.21(122.90)	21.3%	12.8%	20.6%	17.4%
	Tocantins	14.32(0.90)	84.82(110.71)	21.4%	11.9%	23.8%	20.5%
Northeast	Maranhão	14.25(0.93)	81.57(112.25)	25.5%	12.2%	22.9%	12.5%
	Piauí	14.30(1.02)	65.29(102.90)	29.3%	13.4%	19.4%	13.9%
	Ceará	14.28(0.88)	80.02(115.55)	29.4%	11.0%	18.6%	11.7%
	Rio Grande do Norte	14.45(0.98)	76.20(106.38)	27.5%	11.3%	22.8%	14.2%
	Paraíba	14.27(1.03)	73.90(107.51)	26.3%	11.6%	21.9%	17.1%
	Pernambuco	14.22(0.96)	100.56(123.62)	22.8%	11.3%	23.2%	18.0%
	Alagoas	14.37(1.00)	83.91(115.91)	33.0%	12.0%	16.5%	15.1%
	Sergipe	14.41(1.09)	65.11(100.75)	29.9%	11.9%	21.2%	14.2%
	Bahia	14.55(1.08)	92.53(122.88)	26.6%	12.1%	25.0%	13.1%
Southeast	Minas Gerais	14.26(0.75)	90.30(115.97)	19.0%	11.3%	21.7%	17.9%
	Espírito Santo	14.30(0.86)	79.77(101.84)	18.3%	11.5%	24.5%	23.1%
	Rio de Janeiro	14.42(0.85)	92.99(114.57)	15.6%	12.5%	29.8%	15.6%
	São Paulo	13.87(0.77)	86.03(114.17)	18.7%	12.6%	24.8%	17.7%
South	Paraná	14.07(0.96)	81.82(109.24)	18.0%	13.1%	24.0%	21.6%
	Santa Catarina	14.29(0.75)	76.27(107.61)	22.0%	11.9%	23.9%	17.7%
	Rio Grande do Sul	14.57(0.87)	76.01(102.34)	25.3%	14.9%	23.4%	14.5%
Midwest	Mato Grosso do Sul	14.33(0.96)	83.48(112.63)	22.4%	12.5%	23.5%	21.1%
	Mato Grosso	14.04(0.66)	87.56(128.54)	18.2%	11.1%	23.0%	22.1%
	Goiás	14.19(0.81)	88.74(124.55)	20.4%	12.7%	23.8%	17.3%
	Distrito Federal	14.20(0.80)	74.41(109.51)	17.5%	10.8%	27.4%	25.7%

**Table 2:** Descriptive information (percentages) of school-level variables, per federative units

Region	Federative units	School size		Gymnasium		Playground area		Sports equipment	
		≤500 students	> 500 students	There is	Does not exist/ unusable	There is	Does not exist/ unusable	There is	Does not exist/ unusable
North	Rondônia	21.5%	78.5%	87.2%	12.8%	49.5%	50.5%	96.0%	4.0%
	Acre	24.1%	75.9%	58.4%	41.6%	21.7%	78.3%	94.2%	5.8%
	Amazonas	29.9%	70.1%	57.5%	42.5%	56.0%	44.0%	91.6%	8.4%
	Roraima	43.4%	56.6%	73.8%	26.2%	43.1%	56.9%	58.0%	42.0%
	Pará	22.2%	77.8%	55.0%	45.0%	44.7%	55.3%	84.4%	15.6%
	Amapá	18.0%	82.0%	65.4%	34.6%	27.8%	72.2%	69.5%	30.5%
	Tocantins	37.2%	62.8%	81.5%	18.5%	63.5%	36.5%	96.3%	3.7%
Northeast	Maranhão	42.9%	57.1%	32.9%	67.1%	42.6%	57.4%	79.4%	20.6%
	Piauí	56.4%	43.6%	50.3%	49.7%	52.7%	47.3%	87.5%	12.5%
	Ceará	41.2%	58.8%	59.1%	40.9%	36.3%	63.7%	97.9%	2.3%
	Rio Grande do Norte	27.2%	72.8%	45.3%	54.7%	51.8%	42.8%	88.2%	11.8%
	Paraíba	24.7%	75.3%	58.9%	41.1%	44.5%	55.5%	90.8%	9.2%
	Pernambuco	19.8%	80.2%	65.4%	34.6%	50.8%	49.2%	91.7%	8.3%
	Alagoas	19.2%	80.8%	47.6%	52.4%	51.0%	49.0%	88.0%	12.0%
	Sergipe	22.1%	77.9%	42.9%	50.8%	51.4%	47.5%	88.8%	11.2%
	Bahia	29.6%	70.4%	65.4%	34.6%	56.9%	43.1%	93.3%	6.7%
Southeast	Minas Gerais	19.3%	80.7%	84.8%	15.2%	52.3%	47.7%	88.5%	11.5%
	Espírito Santo	19.0%	81.0%	88.0%	12.0%	62.8%	37.2%	98.55%	1.5%
	Rio de Janeiro	20.8%	78.4%	90.6%	9.4%	90.9%	39.1%	97.1%	2.9%
	São Paulo	13.0%	87.0%	94.6%	5.4%	43.2%	56.8%	97.3%	2.7%
South	Paraná	14.1%	85.9%	91.8%	8.2%	50.7%	47.4%	94.9%	5.1%
	Santa Catarina	35.1%	62.8%	85.8%	12.1%	58.1%	39.7%	89.7%	8.2%
	Rio Grande do Sul	31.3%	68.7%	83.5%	16.5%	77.9%	22.1%	96.8%	3.2%
Midwest	Mato Grosso do Sul	15.7%	84.3%	87.7%	12.3%	65.3%	4.7%	96.3%	3.7%
	Mato Grosso	19.4%	80.6%	73.0%	27.0%	44.3%	55.7%	92.3%	7.7%
	Goiás	25.0%	75.0%	77.0%	23.0%	63.3%	36.7%	93.4%	6.6%
	Distrito Federal	5.2%	92.4%	88.1%	9.5%	59.2%	38.3%	95.6%	2.0%

Regarding the school context, it was observed that the majority of them have more than 500 students enrolled. Taking into account the other school context variables, a wide difference was observed across the states, but some similarities were seen within geographical regions.

**Fig. 1:**
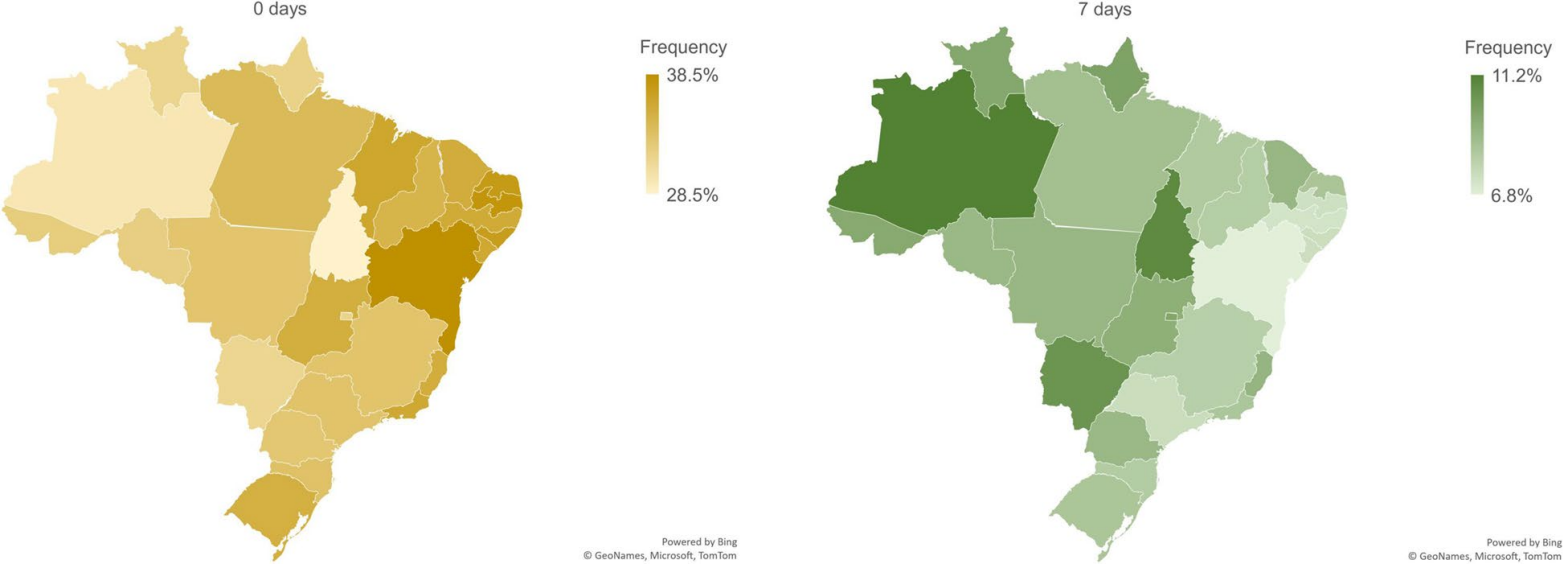
Compliance of physical activity guidelines, per Federative units

Figure 1 shows daily physical activity guidelines compliance, per federative units, indicating percentages of children who comply the guidelines during all the 7 days, as well as those who did not comply in any of the days. In the most of the states, more than 1/3 of the children do not meet the guidelines on any given day during the week. Further, regarding the achievement of the physical activity recommendation in the whole week, in only three federative units (Amazonas, Tocantins, Mato Grosso do Sul) more than 10% of the children meet the guidelines.

Multilevel modelling results are presented in Table 3. Model 1 only included states, where the state of Bahia was chosen as the reference given that it showed the lowest percentage of children who complied with the physical activity recommendations in the whole week (6.8% of the children). Interestingly, it is important to note that all states from the Northeast region, but Rio Grande do Norte, did not present statistically significant differences for Bahia, while the other states significantly differ from it.

**Table 3:** Summary results of mixed models for the number of days of physical activity guidelines compliance

Parameters	Model 1			Model 2			Model 3		
	β (se)	OR	95% CI OR	β (se)	OR	95% CI OR	β (se)	OR	95% CI OR
Fixed effects									
Intercept (Bahia)	−0.924 (0.035)*	---	---﻿	−1.427 (0.038)*	---﻿	---﻿	–1.428 (0.046)*	---﻿	---﻿
Pernambuco	0.055 (0.047)	1.056	0.963-1.158	0.014 (0.051)	1.014	0.917–1.122	0.018 (0.051)	1.018	0.921–1.125
Paraíba	–0.002 (0.046)	0.998	0.913–1.091	−0.015 (0.050)	0.985	0.893–1.086	−0.012 (0.050)	0.988	0.897–1.089
Sergipe	0.069 (0.047)	1.072	0.978–1.174	0.116 (0.051)*	1.123	1.017–1.242	0.122 (0.051)*	1.130	1.022–1.249
Alagoas	0.024 (0.051)	1.024	0.927–1.131	0.026 (0.055)	1.027	0.921–1.144	0.036 (0.055)	1.037	0.930–1.155
São Paulo	0.148 (0.050)*	1.160	1.051–1.279	0.117 (0.055)*	1.124	1.010–1.251	0.116 (0.055)*	1.123	1.009–1.251
Minas Gerais	0.173 (0.046)*	1.189	1.087–1.300	0.175 (0.050)*	1.191	1.080–1.314	0.175 (0.050)*	1.191	1.080–1.314
Piauí	0.086 (0.046)	1.089	0.995–1.193	0.086 (0.051)	1.090	0.987–1.204	0.079 (0.051)	1.082	0.980–1.195
Santa Catarina	0.154 (0.047)*	1.167	1.064–1.280	0.147 (0.052)*	1.159	1.047–1.282	0.147 (0.052)*	1.158	1.046–1.282
Maranhão	0.030 (0.047)	1.031	0.941–1.129	0.025 (0.051)	1.026	0.928–1.134	0.026 (0.051)	1.027	0.928–1.135
Rio de Janeiro	0.132 (0.048)*	1.141	1.039–1.254	0.097 (0.052)	1.102	0.995–1.221	0.088 (0.052)	1.092	0.986–1.211
Rio Grande do Sul	0.117 (0.050)*	1.125	1.021–1.239	0.091 (0.054)	1.096	0.985–1.129	0.087 (0.054)	1.091	0.981–1.214
Rio Grande do Norte	0.104 (0.046)*	1.110	1.014–1.215	0.111 (0.050)*	1.117	1.012–1.233	0.115 (0.050)*	1.122	0.017–1.238
Pará	0.111 (0.049)*	1.117	1.014–1.230	0.101 (0.054)	1.106	0.996–1.228	0.107 (0.054)*	1.113	1.002–1.236
Paraná	0.208 (0.050)*	1.231	1.117–1.357	0.156 (0.054)*	1.169	1.051–1.299	0.154 (0.054)*	1.167	1.049–1.298
Rondônia	0.177 (0.049)*	1.193	1.084–1.313	0.173(0.053)*	1.189	1.071–1.321	0.171 (0.053)*	1.186	1.068–1.317
Mato Grosso	0.184 (0.050)*	1.203	1.090–1.327	0.150 (0.055)*	1.162	1.044–1.294	0.152 (0.055)*	1.165	1.046–1.296
Ceará	0.085 (0.048)	1.089	0.991–1.196	0.094 (0.053)	1.099	0.991–1.218	0.088 (0.053)	1.092	0.985–1.210
Espírito Santo	0.178 (0.046)*	1.194	1.092–1.306	0.133 (0.050)*	1.143	1.036–1.260	0.133 (0.050)*	1.142	1.036–1.260
Goiás	0.207 (0.045)*	1.229	1.126–1.343	0.166 (0.049)*	1.181	1.073–1.300	0.165 (0.049)*	1.180	1.072–1.299
Acre	0.137 (0.050)*	1.147	1.041–1.264	0.123 (0.054)*	1.130	1.016–1.258	0.126 (0.055)*	1.134	1.019–1.262
Roraima	0.289 (0.050)*	1.335	1.212–1.471	0.272 (0.054)*	1.313	1.181–1.460	0.270 (0.055)*	1.310	1.176–1.459
Distrito Federal	0.273 (0.060)*	1.314	1.168–1.477	0.221 (0.065)*	1.247	1.099–1.416	0.219 (0.065)*	1.245	1.096–1.415
Amapá	0.236 (0.049)*	1.266	1.151–1.393	0.207 (0.053)*	1.230	1.108–1.365	0.215 (0.054)*	1.239	1.116–1.377
Mato Grosso do Sul	0.308 (0.050)*	1.360	1.234–1.499	0.273 (0.054)*	1.313	1.181–1.460	0.273 (0.054)*	1.314	1.182–1.461
Tocantins	0.328 (0.049)*	1.388	1.261–1.528	0.269 (0.053)*	1.308	1.178–1.453	0.261 (0.053)*	1.298	1.169–1.441
Amazonas	0.348 (0.050)*	1.416	1.283–1.563	0.351 (0.055)*	1.421	1.276–1.583	0.351 (0.055)*	1.421	1.276–1.582
Sex				0.835 (0.006)*	2.306	2.277–2.334	0.835 (0.006)*	2.305	2.277–2.334
Age (years)				0.043 (0.004)*	1.044	1.037–1.051	0.043 (0.004)*	1.044	1.036–1.051
Mother schooling (elementary school or incomplete high school)				0.095 (0.010)*	1.099	1.078–1.121	0.094 (0.010)*	1.099	1.078–1.120
Mother schooling (high school or incomplete undergraduate)				0.151 (0.009)*	1.164	1.144–1.183	0.153 (0.009)*	1.165	1.146–1.185
Mother schooling (undergraduate or higher level)				0.314 (0.010) *	1.369	1.342–1.395	0.314 (0.010)*	1.369	1.343–1.396
Active travelling to/from school (min)				0.001 (0.000)*	1.001	1.0009–1.001	0.001 (0.000)*	1.001	1.001–1.001
Gymnasium							0.021 (0.017)	1.021	0.989–1.055
Playground area							−0.003 (0.015)	0.997	0.969–1.026
Sports equipment							0.018 (0.025)	1.018	0.970–1.068
School size							−0.044 (0.015)*	0.957	0.928–0.986
**Random effects**									
Intercept	0.0981 (0.0033)			0.1124 (0.004)			0.1116 (0.004)		
− 2LogLikelihood	546,613.78029			389,377.33839			388,068.96220		

*OR* Odds Ratios

*p < 0.05

In Model 2, child variables were included, and it fits the data significantly better than Model 1 (− 2 LogLikelihood for Model 1 = 546,613.78029; − 2 LogLikelihood for Model 2 = 389,377.33839; Δ = 157,236.4419, with 6 df, and *p* < 0.001). It was possible to observe that all the variables included in the model were significant in the explanation of children differences in physical activity compliance rate. Children from Sergipe differ from those from Bahia, complying more the guidelines (OR = 1.123); on the other hand, those from Rio de Janeiro, Rio Grande do Sul, and Pará did not differ from their Bahian peers. On average, boys complied more than girls (OR = 2.306), as well as older children (OR = 1.044), and those who spent more time in active travelling/week to/from school (OR = 1.001) complied more than their youngest and those who spent less time in active travelling to school peers, respectively. In addition, increasing mother educational level, increased the odds of children compliance of the physical activity guidelines.

In the Model 3, the school-variables were included, namely school size, and the existence of gymnasium, playground area, and sports equipment. This last model fits the data better than Model 2 (− 2 LogLikelihood of Model 2 = 389,377.33839; − 2 LogLikelihood of Model 3 = 388,068.96220; Δ = 1308.37619, with 4 df, *p* < 0.001). Among states, results were similar from those observed in model 2. The child-level variables remained significant, with small changes, meaning that their interpretation did not differ to that from the previous model. Further, from the school context variables, only school size showed to be a significant parameter for children compliance guidelines, where children enrolled in schools with more than 500 students were less likely to comply with the physical activity guidelines (OR = 0.957).

## Discussion

This study aimed to verify differences between the Brazilian federative units regarding to youth physical activity guidelines accomplishment and to investigate the determinants related to these differences. The results demonstrated that only in three states more than 10% of the children were active at all the 7 days (Amazonas, Tocantins, and Mato Grosso do Sul). Notwithstanding data from the present study comes from one single country, it is observed a range variability in children physical activity compliance between states; although in the present study it was not explored states characteristics that could explain these differences, it is possible that these results may be related to differences in socioeconomic, cultural, and political characteristics [16], that may influence children involvement in physical activities. These aspects were previously reported in a multinational study, where it was demonstrated the role of social disparities on children’s physical activity difference across countries [14].

Results of the multilevel analysis revealed differences in physical activity compliance rate across states. The Bahia site was the reference (its adolescents had the lowest compliance rate), and the States-Model (Model 1) revealed that within Northeast region, only adolescents from the Rio Grande do Norte state complied significantly more than Bahian youth, with no other significant difference observed among others Northeastern states. It is well knowing that physical activity is influenced by socio-environmental aspects [17], such as crime rates and individual perception of safety [18]. For example, Lopes et al. [19] reported that a positive perception about the neighbourhood (perception of safety, neighbourhood aesthetics, and an attractive environment) was associated with higher involvement in physical activity by youth, despite of differences in this perception were observed, which may explain differences in physical activity levels between sex. Further, Janssen [20] studying a large sample of adolescents, concluded that both, perception of safety and crime rate, were related to youth physical activity outside school, where decreasing the perception of safety and/or increasing the neighbourhood crime, leads to decreasing of physical activity. Taking this into account, in the Brazilian context, according to data from the Institute of Applied Economic Research [21], the Northeast region presents the highest crime index in the country, and when this information is presented by states, Bahia site is one of the most violent states, which can explain the lowest compliance rate observed among its adolescents, and also the similarity in results found among other seven Northeastern states (except for Rio Grande do Norte).

There is a consensus about the influence of biological and demographic factors on physical activity levels, which was explored in the model 2. Regarding to child-level variables explored in the present study, all of them showed to be significant predictors of youth physical activity compliance rate. Older youth complied more the guidelines than their youngest peers, and this result differs from some of those previous reported, where authors suggest that  both biological (such as hormones release) and environmental characteristics (behaviours and responsibilities acquired with age, such as family, work, studies) can lead to the reduction of physical activity over time [22, 23]. Regarding sex role on physical activity, Nader et al. [24] for example, studying youth aged 9-15 years, reported that sex is a relevant determinant for physical activity, where boys were more active than girls; similarly, other studies have also reported this sex dimorphism in physical activity [25, 26], and it seems that this fact may be related to some behavioural and social aspects that favour boys in their involvement in physical activity and sports participation, allowing them to be more physically active than girls [27].

Further, it is known that parents/legal guardians socioeconomic status may play a relevant role in children's physical activity involvement [28] and legal guardian educational level (specially mother educational level), as well as family income, has being used as socioeconomic determinants [29]; however, there is no consensus about this relationship. For example, Sherar et al. [30] showed that girls whose mothers reported low educational level were less physically activity, while Vazquez-Nava et al. [31] and Ball et al. [32] found the opposite, i.e., youth whose mothers had higher educational level were those with the lowest physical activity level. In the present study, mother educational level was a significant predictor for youth physical activity compliance, where increasing maternal educational level, the odds of youth being physically active also increased, which can be related to the fact that mothers with higher educational level have more information about healthy eating and physical activity habits [31], that can be reflected in a more supportive attitudes for encourage their children to get involved in physical activity [33].

Since children spend a relevant part of their awake time at school, this environment seems to be of relevance for students' physical activity, since most of their active and healthy habits can be created and/or maintained in it [34]. For example, from the variables of the school context, Chaves et al. [35] suggest that school variables explain approximately 10% of the variance in children's motor coordination development, being able to act as a mediator for physical activity [36]. In the present study, only school size was significant in the explanation of children's differences in physical activity compliance, where those children from the smallest schools (with fewer students) were more active during the whole week than their peers from the biggest ones. It is possible that this “smallest” environment may be more conducive to the development of physical activities, because there are more free places (children per m^2^) where these activities can be performed by students [35, 37].

This study has some limitations, namely (1) the use of questionnaire to collect information regarding youth physical activity/active commuting, which can be prone to error and/or bias in information; however, epidemiological studies have used this approach to data collection, meaning that this is a usual strategy used when large number of subjects are sampled, given the positive cost-effectiveness relationship; (2) the limited number of school predictors used could not be enough to better understand the role of school context on Brazilian youth physical activity, but the variables used were selected according to previous studies where the role of school on scholars’ physical activity was investigated; furthermore, previous studies have also reported a set of school variables that were not significant in the explanation of differences in youth physical activity level; 3) the model's goodness was assessed using the cross-validation procedure and, based on it, the results found in the present study cannot be generalized for the entirely Brazilian school-age adolescents. But the study has some strengths, such as the use of a large sample size, with youth from all the Brazilian federative units, with different socioeconomic and cultural characteristics; the use of multilevel analysis, allowing to understand the complex issue of individual- and school-level variables and how they can influence on youth physical activities practice.

## Conclusions

It was possible to observe that most of Brazilian schoolchildren did not comply with the daily physical activity guidelines, with a large variance across the states, where Bahian children were those who complied the least, while their peers from Amazonas were those who complied the most. Boys, older youth, and those who spent more time in active travelling per week to/from school were more likely to achieve the physical active guidelines, as well as it was also observed a significant role of maternal education level on it. Moreover, from the set of school variables, only school size (namely the number of students enrolled in school) was a significant predictor to physical activity achievement. The results highlight the role of individual and environmental characteristics (states and school) in children's daily physical activity compliance, and especially in the Brazilian context it shows that states constraints play a relevant role in these differences, meaning that policies should be thought taking into account these environmental discrepancies.

## Data Availability

Data are disponible in: https://www.ibge.gov.br/estatisticas/sociais/populacao/9134-pesquisa-nacional-de-saude-do-escolar.html?=&t=downloads.
